# The Implications of Pruritogens in the Pathogenesis of Atopic Dermatitis

**DOI:** 10.3390/ijms22137227

**Published:** 2021-07-05

**Authors:** Lai-San Wong, Yu-Ta Yen, Chih-Hung Lee

**Affiliations:** 1 Department of Dermatology, Kaohsiung Chang Gung Memorial Hospital and Chang Gung University College of Medicine, Kaohsiung 833, Taiwan; laisan7@hotmail.com; 2 Department of Dermatology, Fooying University Hospital, Pingtung 928, Taiwan; camm.tw@yahoo.com.tw

**Keywords:** itch, atopic dermatitis, pruritogens

## Abstract

Atopic dermatitis (AD) is a prototypic inflammatory disease that presents with intense itching. The pathophysiology of AD is multifactorial, involving environmental factors, genetic susceptibility, skin barrier function, and immune responses. A recent understanding of pruritus transmission provides more information about the role of pruritogens in the pathogenesis of AD. There is evidence that pruritogens are not only responsible for eliciting pruritus, but also interact with immune cells and act as inflammatory mediators, which exacerbate the severity of AD. In this review, we discuss the interaction between pruritogens and inflammatory molecules and summarize the targeted therapies for AD.

## 1. Introduction

Pruritus is defined as a desire to scratch, which is a defense mechanism to eliminate possible noxious stimuli; however, it may damage the skin barrier. Atopic dermatitis (AD) is a chronic inflammatory disease characterized by pruritus, and is recognized as a prototype of chronic pruritic dermatosis [1].

The pathophysiology of AD is complex, involving several mechanisms. Impairment of skin barrier function; and dysregulation of T helper (Th) 2 and Th1 cytokines are some of the major pathogenic components involved in AD. Skin barrier disruption induces the release of large amounts of keratinocyte-derived proinflammatory cytokines such as IL-33 and TSLP, from keratinocytes. It also activates the activation of the inflammatory cascade, mainly involving Th2 cytokines such as IL-4, IL-13, and IL-5 [2]. Notably, various pruritogens, including proteases and some of the inflammatory cytokines mentioned above, are produced simultaneously and, in turn, activate the cutaneous nerve fiber terminals, which are widely distributed over the skin [3]. An itch is predominantly mediated by unmyelinated C afferent fibers and a small amount of myelinated Aδ afferent fibers originating from the dorsal root ganglion (DRG) [4]. The excited free nerve endings not only transmit itch signaling by afferent sensory neurons but also release various neuropeptides from efferent nerve fibers, stimulating immune cells such as mast cells (MCs), Th2 cells, and eosinophils. The mediators from immune cells reciprocally activate primary afferent neurons [5]. Moreover, scratches triggered by an itch sensation may further damage the skin barrier, and can initiate a vicious itch-scratch-inflammation cycle. Therefore, pruritogens, substances eliciting the sensation of an itch, are a cause and effect of AD [3].

Histamine is the first and most well-known pruritogen [6]. A large volume of evidence implicating other mediators may be involved in the pathophysiology of itch response during AD [2]. Surprisingly, some of the inflammatory mediators, such as IL-4/IL-13, which were previously thought to induce itch indirectly by promoting pruritogens released from immune cells, may induce itch directly by activating sensory neurons [7,8]. Accordingly, based on the histamine axis, the molecular pathway of pruritus can be classified as histamine-dependent and -independent signaling. Several studies have shown that pruritus in AD largely relies on histamine-independent signaling since antihistamine medication cannot control pruritus in AD [2]. Currently, many emerging therapies targeting pruritogens are being developed to treat intractable itching and chronic inflammation in AD (Table 1).

**Figure 1:**
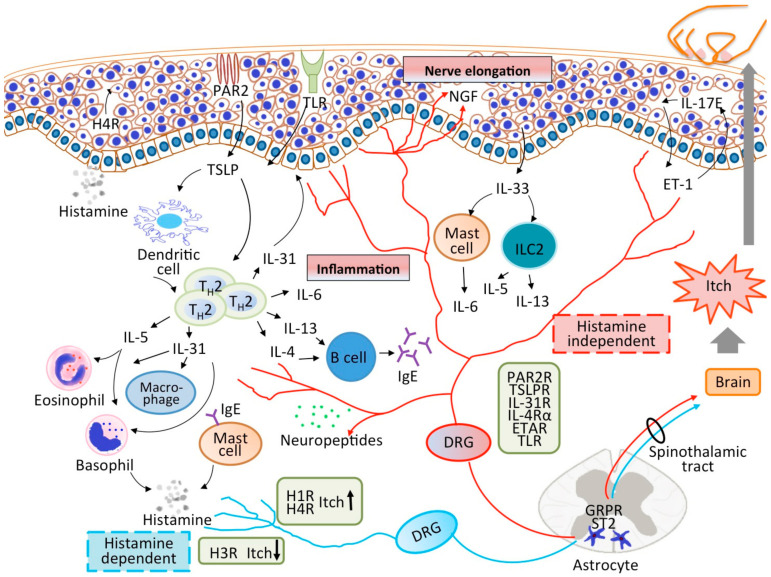
The interplay among the skin barrier, immune system, and nervous system in patients with AD. Skin barrier disruption promotes the release of inflammatory cytokines from keratinocytes, leading to immune activation, particularly of the Th2 pathway. Th2 cytokines initiate the inflammatory process by recruiting and activating more immune cells to the dermis. Simultaneously, various pruritogens are released, activating the cutaneous nerve fiber terminals. The itch sensation can be transmitted by histamine-dependent or histamine-independent pathways. Neuropeptides are released by efferent fibers and stimulate the immune cells to exacerbate the inflammatory process. Moreover, scratching triggered by an itch may further damage the skin barrier, leading to a vicious cycle.

From the clinical and physiological aspects, this review aims to focus on the interaction between pruritogens and inflammation in the pathophysiology of AD (Figure 1).

## 2. Histamine-Dependent Pruritogens

### 2.1. Histamine

Histamine is released primarily by MCs and basophils during allergic inflammation and exerts multiple functions via four receptors (H1R-H4R) [6]. It is well recognized that histamine induces itch and activation of H1R and H4R, while inhibition of H3R can excite sensory neurons by inducing Ca^+^ influx [20]. H1R has been well evaluated for its significant role in acute itch such as urticarial diseases; however, antihistamine-targeting H1R has limited efficacy in most AD-related pruritus [21]. On the other hand, histamine also regulates sweating, which is commonly impaired in patients with AD. Sweat glands also express histamine receptors, and H1R can abolish acetylcholine (ACh) mediated sweating [22]. This suggests that the interaction between H1R and ACh receptors in sweat glands may regulate the pathophysiology of AD. In contrast, H4R has been the focus of recent studies; it has been found to be predominantly expressed in immune cells, as well as in keratinocytes and sensory neurons [23,24,25]. It has been demonstrated that the expression of H1R and H4R mRNA is higher in AD lesioned keratinocytes than in patients with psoriasis and healthy individuals. Furthermore, the promotion of keratinocyte proliferation, which is common in chronic eczematous changes in AD, was contributed by H4R in patients with AD but not in patients with psoriasis [24]. Consistent with this, Rossbach et al. showed a reduction in inflammatory cells and epidermal hyperproliferation in H4 knockout mice challenged with ovalbumin [26]. It has been reported that the expression of H4R in Th2 cells is upregulated in patients with AD and can be enhanced by IL-4. H4R agonists can induce IL-31, an important inflammatory cytokine and pruritogen, in peripheral blood mononuclear cells (PBMCs) from patients with AD [23]. H4R also upregulates thymic stromal lymphopoietin (TSLP) from human keratinocytes, a crucial epithelial-derived cytokine in AD [27]. A recent study found that histamine increased the expression of oncostatin M (OSM), a proinflammatory mediator involved in the pathogenesis of psoriasis and AD, in M1 macrophages via the activation of H1R, H2R and H4R, and in turn, OSM stimulated STAT3 phosphorylation in human keratinocytes [28]. Notably, the dual inhibitory effects of the H4R antagonist on both pruritus and Th2 inflammatory responses were demonstrated in an allergic contact dermatitis mouse model [29]. In line with these findings, we suggest that H4R may mediate pruritus directly on sensory neurons and indirectly by modulating well-known pruritogens, such as TSLP and IL-31. Notably, combination therapies of H1R and H4R antagonists showed synergic anti-inflammatory action and were more beneficial than the H1R or H4R monotherapies in an AD mouse model [30]. In addition, it has been shown that epinastine, an H1R antagonist, decreases serum IL-31 levels in patients with AD [31], which may indicate a possible cross-reaction between H1R and H4R. JNJ 39758979, a potent and selective H4R antagonist, significantly reduced histamine-related pruritus in healthy individuals [32]. A phase 2a study conducted in Japan showed significant improvement in pruritus with JNJ39758979 at oral dosages of 100 mg and 300 mg in Japanese adults with moderate AD; however, further studies were terminated due to drug-related agranulocytosis [9]. Another oral H4R antagonist, ZPL-3893787, significantly decreased EASI, SCORED, and IGA 0/1 scores in a randomized, double-blind phase 2 trial in adults with moderate to severe AD. Nevertheless, ZPL-3893787 attenuated pruritus without statistical significance [10].

### 2.2. Platelet-Activating Factor (PAF)

PAF is a lipid inflammatory mediator that can be released from many immune cells, including eosinophils, MCs, neutrophils, basophils, and epithelial cells [33]. PAF amplifies the immune response by triggering degranulation, chemotaxis, and adhesion of immune cells, such as eosinophils and MCs [34]. Intradermal injection of PAF can induce wheal and itch, which is related to histamine release from MCs via a neurogenic response because it can be blocked by nerve blocks [35,36,37]. A post hoc analysis in a Japanese study showed that rupatadine, an H1 antihistamine with a PAF antagonist improved total pruritus score in patients with AD [11].

## 3. Histamine-Independent Pruritogens

### 3.1. Protease and Protease-Activated Receptors (PARs)

Protease is important for skin barrier homeostasis. Aberrant protease activity contributes to the disruption of the skin barrier and, in turn, induces downstream inflammatory cytokines through the activation of the corresponding receptors [38,39]. The numerous activities of proteases are mediated by PARs. PARs are G protein-coupled receptors and consist of four members: PAR1, PAR2, PAR3, and PAR4. They can be activated by proteases endogenously from keratinocytes and immune cells and exogenously from dust mites, *S. aureus*, and fungi, all of which are essential triggers of AD [40]. Among the four receptors, the functional roles of PAR2 have been more clearly described in AD [38]. Protease activity and PAR2 expression in keratinocytes and PAR+ peripheral nerves were prominently increased in lesioned skin of AD patients [41,42]. PAR2 can be activated by specific serine and cysteine proteases such as tryptase, kallikreins (KLK), and cathepsin. It has been demonstrated that KLK5 activates PAR2 to induce nuclear factor κB-mediated overexpression of TSLP in keratinocytes [39,43]. However, recent studies have shown that KLK5 induced AD-like skin architecture changes in a PAR2 independent manner and that KLK7 promotes an itch response independent of skin inflammation and PAR2 [44,45]. This suggests that non-PAR2 mediated responses are also important in the pathogenesis of AD. Recently, Zhao et al. demonstrated that TRPV3, a warm-temperature-sensitive channel in keratinocytes, modulates PAR2-associated cytokine production, such as TSLP, indicating that PAR2-TRPV3 signaling in keratinocytes play a role in inflammation and pruritus transmission in AD [46]. Two groups have recently shown the regulatory function of neuronal alteration by PAR2 in keratinocytes in a Grhl3^PAR/+^ mouse-model overexpressing PAR2 in suprabasal keratinocytes. Spontaneous AD-like dermatitis and increased scratching behavior were found in these mice, and skin inflammation was exacerbated by house dust mite (HDM) protease, a major source of allergens in AD [47]. However, another group showed that HDM challenge is necessary for AD-like dermatitis and increased scratching behavior [48]. The phenotypic differences may be caused by the various genetic backgrounds, age of the mice, and housing conditions. Subclinical skin barrier impairment was observed in Grhl3^PAR/+^ mice and increased nerve fiber density after HDM challenge [47]. Importantly, PAR2 expression in the DRG was upregulated even without HDM challenge in mice overexpressing epidermal PAR2 [47]. In addition, skin disruption in HDM-treated Grhl3^PAR/+^ mice also upregulated pruritus and inflammatory-related gene expression in sensory neurons, including TSLPR, IL-31 receptor α, and brain-derived neurotrophic factor (BDNF) [47,48]. Evidence supports neuro-epidermal communication by PAR2 in keratinocytes. PAR2 is located not only in keratinocytes and peripheral nerves but also in primary sensory neurons. The functional roles of PAR2 in keratinocytes and DRG may differ [49]. Some proteases can activate PAR2+ sensory neurons directly or sensitize other channels, such as TRPV1 channels, which might contribute to non-histaminergic itch [50]. For instance, it has been shown that cathepsin S, a cysteine protease, acts via PAR2 activation in TRPV1 sensory neurons to induce scratching behavior in mice [51]. PZ-235, a PAR2 pepducin, suppressed PAR2-mediated inflammation and itch response in an AD mouse model [52]. Topical E6005, a phosphodiesterase 4 inhibitor, showed an antipruritic response, which is associated with the PAR2 pathway [53]. More clinical trials in humans are required to uncover the inflammatory and neuronal functions of PAR2 in AD.

### 3.2. Thymic Stromal Lymphopoietin (TSLP) and TSLP Receptor (TSLPR)

TSLP is a keratinocyte-derived cytokine with two variants: short- and long-form [54,55]. Short-form TSLP is involved in homeostasis while long-form TSLP is responsible for the proinflammatory response [56,57]. TSLP signals via a TSLP receptor (TSLPR), a heterodimer of the IL-7 receptor α chain and the TSLPR chain [58]. TSLP is highly expressed in the lesioned skin and serum of AD patients [59]. TSLP can be triggered by environmental allergens, microbes, and endogenous inflammatory mediators, such as proteases and Th2 cytokines [60]. TSLP is known to act on dendritic cells (DCs) in the skin, inducing functional Th2 cell differentiation [61]. Recently, it has been reported that TSLP may induce T follicular helper cells, which are related to the severity and pathogenesis of AD and differentiation, by activating both DCs and Langerhans cells (LCs) [62,63]. It has been shown that higher expression of TSLPR on CD4+ T cells in patients with AD and TSLP can activate Th2 cells to promote IL-4 expression [64]. In addition, TSLP can drive Th2 cells into having a pathogenic phenotype, which promotes greater amounts of IL-5 and IL-13 compared to non-TSLP-treated Th2 cells [65]. TSLP has been demonstrated to enable the development of a population of IL-13+ single-positive CD4+ T cells with expression of Th2 effector cytokines [66]. Direct induction of dermal T cell migration in the absence of DCs has been demonstrated [67]. In line with the above evidence, this indicates direct interaction between TSLP and T cells. Furthermore, TSLP activates various immune cells, such as eosinophils and B cells [54]. However, there are some discrepancies among the capacities of TSLP for the activation of human basophils [68,69]. Moreover, TSLP can disrupt the skin barrier by downregulating filaggrin (FLG) expression, a key protein in the development and maintenance of the skin barrier via the STAT3 and ERK pathways [70]. It is noteworthy that TSLP released from keratinocytes can exacerbate allergy-induced asthma and promote “atopic march” (the progression of atopic disorder to the development of allergy-induced rhinitis and asthma) [71]. TSLP evokes immediate pruritus when TSLP is injected into the cheek of mice. In addition, ORAI1/NFAT signaling-related TSLP release by keratinocytes activates TSLPR in the DRG evoking TRPA1-dependent itch behavior in mice [8]. In a phase 2a randomized double-blinded study, tezepelumab (AMG 157, MED19929), a humanized anti-TSLP antibody, showed a trend towards improvement in pruritus and severity of AD at week 16 in combination with topical corticosteroids (TCS) in moderate to severe AD [12]. A phase 1b study using MK8226, an inhibitor of the TSLP receptor, was also conducted in patients with moderate to severe AD; however, the study was terminated for business reasons.

### 3.3. IL-33

IL-33, a member of the IL-1 cytokine family, is constitutively expressed at the protein level in the nuclei of endothelial and epithelial cells. It acts as an alarmin, which can be triggered by skin barrier damage or pathogen provocation, and is rapidly released from the nucleus of keratinocytes. IL-33 signals via its receptor, ST2 (also known as IL-33R α chain or IL-1RL1), which is heavily expressed in MCs and is also expressed in Th2 cells, group 2 innate lymphoid cells (ILC2s), and basophils [72,73,74]. Increased expression of ST2 was found in lesioned skin of patients with AD, and the expression of ST2 and IL-33 was upregulated after exposure to allergens and Staphylococcus enterotoxin B [75]. Kindi et al. also showed that *Staphylococcus aureus* secondary immunoglobulin-binding protein can induce IL-33 release from keratinocytes [76]. Increased serum IL-33 levels have been reported in patients with AD, and are correlated with the severity of AD [77]. Another group also reported that the expression of IL-33 in keratinocytes was correlated with the severity of pruritus and the degree of lichenification (thick and leathery skin) in patients with AD [78]. IL-33 is a potent MC activator and can stimulate ILC2s and basophils to release Th2 cytokines such as IL-5 and IL-13, which in turn exacerbate inflammation in AD [79,80]. Transgenic mice with IL-33 overexpression in keratinocytes showed AD-like inflammation, which is dependent on the innate immune response mediated by ILC2s and basophils [80]. In addition, IL-33 induces and maintains the Th2 inflammatory response by enhancing the function of the TSLP-DC-OX40L axis [81]. IL-33 can disrupt skin barrier function by downregulating FLG expression via the STAT3 and ERK pathways [82]. Liu et al. showed that ST2 is present in the free nerve endings of the skin and primary sensory neurons of humans and mice, of which the ST2+ neurons are coexpressed with TRPV1+ neurons. Furthermore, IL-33 can excite DRG neurons by inducing Ca^+^ influx. Neutralized antibodies against IL-33 or ST2 reduced skin inflammation and pruritus in a mouse model of poison ivy allergic contact dermatitis. Knockdown of ST2 expression in DRG neurons can attenuate both scratching and skin inflammation, indicating the interaction of the neurocutaneous system [83]. On the other hand, Du et al. showed that spinal IL-33/ST2 signaling by astrocytic JAK2-STAT3 cascades, which promoted TNF-α to sensitize gastrin-releasing peptide (GRP)-GRP receptor (GRPR) signaling, participated in IL-33/ST2 mediated chronic itch. ST knockout mice can attenuate scratching in a 2,4-dinitrofluorobenzene (DNFB) induced allergic contact dermatitis mouse model and dry skin mouse model [84]. A phase 2a proof-of-concept clinical trial of etokimab (ANB020), a humanized monoclonal antibody against IL-33, revealed a rapid response with EASI 50 of 83% and EASI 75 of 33% by day 29 with a single-dose injection in 12 adults with moderate to severe AD, and the response was sustained at day 57. Improvements in the DLQI and 5D itch scales were also significant [13]. The phase 2b trial of etokimab (ATLAS trial) is currently under evaluation. Another agent targeting IL-33, REGN3500, as a monotherapy or in combination with dupilumab, has completed the phase 2 trial, but the results have not been released. Similarly, PF-06817024 targeting IL-33 has completed a phase 1 trial; however, the results are not available.

### 3.4. IL-4 and IL-13

IL-4 and IL-13 are both pivotal type 2 inflammatory cytokines in allergic diseases including AD [85]. IL-4 signals via two types of receptors and the expression of these two receptors vary in different cells. Type 1 receptors are composed of the IL-4 receptor α chain and the common γ chain, and the type 2 receptor is composed of the IL-4 receptor α chain and IL-13 receptor α1 chain. Type 1 and type 2 receptors are distributed mainly in hematopoietic and non-hematopoietic cells, respectively. The IL-13 receptor α1 chain is a ligand subunit for IL-13, and IL-13 also binds to the IL-13 receptor α2 chain, which is primarily expressed in structural cells and fibroblasts [86,87]. Just as IL-4 and IL-13 have shared receptor subunit, IL-4 and IL-13 also shared similar biological activities; however, recent studies indicate that IL-4 is more relevant in Th2 response and humoral immunity, while IL-13 is more crucial in the tissue [88,89]. In addition to the well-known function of IL-4 in amplifying Th2 inflammation and IgE production, a recent study reported that IL-4 enhanced IL-31/IL-31 receptor α signaling, which is important in the pathogenesis and pruritus transmission in AD [90]. Transgenic overexpression of IL-13 in the skin of a mouse can induce a pruritic phenotype [91]. Bitton et al. demonstrated that dermatitis with epidermal and ear thickening was predominantly mediated by IL-13 signaling via type 2 receptors in models of oxazolone- and DNFB-induced dermatitis [92]. It has been suggested that Th2 cytokines are responsible for inflammation in AD, and a study has found that IL-4 receptor α was present in human DRG sensory neurons, which can be directly activated by IL-4 and IL-13. Direct admission of either IL-4 or IL-13 did not induce acute itching; however, it potentiated sensory neurons to multiple pruritogens. Furthermore, the neuronal IL-4 receptor α is crucial not only for chronic itch but also for skin inflammation [7]. Dupilumab, a humanized IL-4 receptor α blocker, has been approved for the treatment of AD and has shown a significant and rapid reduction in both pruritus and severity of AD [93,94]. More biologics targeting the IL-4 receptor have been developed. Pitrakinra completed a phase 2a trial, but the data are not available. Clinical trials of CBP-201, CM310, and AK120, all targeting IL-4 receptors, are currently being conducted. Tralokinumab, a humanized IL-13 specific antibody, demonstrated significant improvement in pruritus and multiple AD severity parameters at week 16 as a monotherapy in a phase 3 trial (ECZTRA 1 trial), and a sustained response was shown at week 52 in the ECZTRA 2 trial [14]. In the ECZTRA 3 trial, tralokinumab was combined with TCS to achieve primary and secondary endpoints [15]. Lebrikizumab, a humanized high-affinity IL-13 antibody, showed a dose-dependent response with significant improvement in AD severity and reduction in pruritus as early as day 2 in the higher-dosage group [16].

### 3.5. IL-31

IL-31, a member of the gp130/IL-6 family, is expressed primarily by Th2 cells and signals via a heterodimeric receptor formed by the IL-31 receptor α chain (IL-31RA) and OSM receptor (OSMR) β chain, which is expressed by immune cells, keratinocytes, cutaneous free nerve endings, and human DRG [95]. IL-31 can be induced by multiple stimuli, such as IL-4 and staphylococcal superantigen [96,97]. A transgenic IL-31 overexpression mouse model showed AD-like skin inflammation and severe scratching behavior [98]. Increased expression of IL-31+ cells in lesioned skin and IL-31RA in both keratinocytes and nerve fibers has been demonstrated in patients with AD [99]. IL-31 mRNA expression is also enhanced in atopic non-lesioned skin [100]. Additionally, a positive correlation between serum IL-31 levels and the severity of AD patients has been reported [101]. Eosinophils are predominantly infiltrated in the dermis of patients with AD can release IL-31, and can be regulated by IL-31 functionally, such as the induction of chemotaxis in eosinophils [102]. IL-31 and IL-33 together could stimulate eosinophils and fibroblasts to produce more AD-related cytokines and chemokines, suggesting the coordinated roles of these cytokines in AD [103]. Recent data showed that basophils are a source of IL-31, which can stimulate basophils to produce proinflammatory cytokines, such as IL-4 and IL-13 [104]. In addition to promoting the inflammatory response, IL-31 can regulate keratinocyte differentiation and skin barrier function. Inhibition of keratinocyte differentiation and downregulation of FLG expression by IL-31 have been reported [105]. Recent studies have demonstrated that IL-31- IL-1 signaling is involved in dysregulated skin barrier function [106]. Increased transepidermal water loss, epidermal thickening, and inflammation with repeated IL-31 administration in the dermis of mice have been reported, indicating the important role of IL-31 in skin barrier remodeling [107]. Cutaneous and intrathecal administration of IL-31 can induce dose-dependent scratching in mice [108]. Meanwhile, increased cutaneous nerve fiber density was found in transgenic IL-31 mice and in the wild-type mice with continuous subcutaneous administration of IL-31. In addition, IL-31 can promote neuronal growth gene expression, activate sensory neurons, and promote sensory nerve elongation and branching. The evidence above supports the critical role of IL-31 in both pruritus and skin hypersensitivity, which are characteristic of AD. IL-31-induced pruritus was reported to be associated with TRPA1 and TRPV1 neurons; however, IL-31-mediated neuronal growth was independent of the TRPV1 channels [108,109]. In contrast to TSLP, IL-31 induced late pruritus response in humans after skin challenge, suggesting that IL-31 may exert pruritus indirectly via secondary mediators from skin cells such as keratinocytes rather than direct activation of sensory nerve receptors in the skin [110]. One study showed that IL-31 augmented the release of brain-derived natriuretic peptide (BNP), which is a crucial peptide for the central itch, from peripheral sensory neurons. In addition, BNP and BNP receptors were upregulated in skin of patients with AD. Meng et al. further showed that BNP can promote AD-related proinflammatory cytokines released from skin cells, thereby IL-31 may regulate AD-related pruritus via BNP signaling [111]. In a phase 2b trial, nemolizumab, a humanized antibody targeting IL-31RA, in combination with TCS, demonstrated significant improvement in the EASI score and pruritus at a dose of 30 mg every 4 weeks [17]. In a phase 3 trial, a greater reduction in pruritus score was demonstrated at a dose of 60 mg of nemolizumab in combination with TCS every 4 weeks in the Japanese AD group [18]. A clinical trial evaluating the long-term efficacy of nemolizumab for moderate to severe AD is currently ongoing. BMS-981164, a humanized IL-31 antibody, completed a phase 1 trial, with no information currently available. A clinical trial was conducted evaluating vixarelimab (KPL-716) targeting the OSMR β chain in other chronic pruritic diseases, and it may also be a potential therapy for AD [112].

### 3.6. IL-6

IL-6, similar to IL-31, is a member of the gp130/IL-6 family and can activate IL4+ CD4+ T cells [113]. IL-6 is secreted by activated T cells [114] and MCs [115] and has been reported to show enhanced expression in the skin [116] and T cells from patients with AD [114]. Tocilizumab (an IL-6 antagonist) showed a decrease compared to the original EASI score of more than 50% in three patients with severe AD, but was associated with bacterial superinfection [117]. The involvement of IL-6 in calcium phosphate-induced pruritus [118] and prurigo nodularis [119] have been reported. Further investigation of the pathological role of IL-6 in AD-related pruritus is necessary.

### 3.7. Endothelin-1 (ET-1)

ET-1, also known as a histamine-independent pruritogen, was originally a potent vasoconstrictor. There are two receptors for ET-1, ETAR and ETBR, with the former being responsible for pruritus transmission. Neuronal endothelin-converting enzyme 1 is a negative regulator for ET-1-mediated pruritus [120]. Increased ET-1 expression in the lesioned skin of patients with AD has also been reported [121]. Plasma ET-1 levels are enhanced and are correlated with serum IgE levels, itch intensity, and the severity of AD [122]. Our group also demonstrated enhanced ET-1 expression in the epidermis and serum of patients with prurigo nodularis, a recalcitrant pruritic dermatosis [123]. ET-1 upregulates IL-25 (IL-17E) via ETAR, and IL-25 reciprocally induces ET-1 from keratinocytes [121]. IL-25 is a strong cytokine mediating type 2 immunity; therefore, the mutual feedback between ET-1 and IL-25 may contribute to the vicious cycle in the pathogenesis of AD [124]. HDM can also induce ET-1 production in keratinocytes via PAR2 [125]. Recently, a study revealed that bosentan, a nonselective ETAR and ETBR antagonist, alleviates dermatitis and pruritus in a mite-induced AD mouse model. It was shown that ET-1, analogous to IL-31, promotes small sensory neuron nerve elongation and branching via the p38 MAPK and JNK pathways, suggesting that ET-1 may contribute to pruritus and skin hypersensitivity in AD [126].

### 3.8. Neurotrophins (NTs)

NTs are a group of neuropeptides, including nerve growth factor (NGF), BDNF, NT-3, and NT-4/5, that signal via high-affinity tropomyosin-related kinase (Trk) cell surface receptors and low-affinity p75^NTR^ cell surface receptors. NTs are known for their role in neuronal survival, function, and development [127]. Several studies have shown that the expression of NGF and NGF receptors (TrkA) in the skin and serum NGF levels increases in patients with AD [128,129]. However, the inconsistent finding showed a lower level or no correlation between serum NGF levels and AD activities in other groups, suggesting a possible local effect rather than a systemic effect of NGF on the pathophysiology of AD [130,131]. NGF can be secreted by various immune cells, but the main source of NGF is keratinocytes in the skin [127]. It is well recognized that enhanced NGF production in the skin of patients with AD plays a role in hyperinnervation, contributing to peripheral sensitization and pruritus in AD [132]. In addition, NGF promotes the survival and activities of MCs, which appear substantially in the early phase of AD [133,134]. Elevated serum BDNF levels have been demonstrated in patients with AD, and serum NGF levels are correlated with eosinophil cationic protein levels and AD severity [135]. Eosinophils are the major source of BDNF in the skin and have an enhanced expression of the BDNF receptors TrkB and p75^NTR^. BDNF induces eosinophil chemotaxis and inhibits eosinophil apoptosis in patients with AD, showing an autocrine signaling process [136]. Additionally, enhanced expression of BDNF+ eosinophils has been shown in the dermis of AD patients, and these eosinophils are located close to the peripheral nerves of the skin. BDNF released from eosinophils in patients with AD has been demonstrated to be a neurite outgrowth factor that mediates the branching of peripheral skin nerves [137]. In a clinical 2b trial, pegcantratinib (CT327), a topical TrkA inhibitor, has been shown to significantly reduce pruritus in patients with psoriasis [138]. More clinical trials are necessary to evaluate the possible role of NTs in AD.

### 3.9. Neuropeptides

Cutaneous sensory nerve terminals can release multiple neuropeptides such as substance P (SP), calcitonin gene-related peptide (CGRP), and vasoactive intestinal peptide (VIP), which can be released following tissue damage and inflammation. In addition to the nervous system, numerous neuropeptides are widely distributed in peripheral tissues and act as crucial immunomodulators. Among these neuropeptides, the most well-described mediators of AD pathogenesis are SP and CGRP [5].

Substance P (SP) is one of the tachykinin neuropeptides, signaling mainly by the neurokinin 1 receptor (NK1R), also known as tachykinin receptor 1 (TACR-1), which is expressed in the central and peripheral nervous system, and a variety of cell types in the skin, including keratinocytes, MCs and immune cells [139]. In addition to NK1R, SP can bind to Mas-related G protein-coupled receptors (MRGPCRs), which are implicated in nociception and itch signaling [140]. Increases in SP expression in serum and lesioned skin and NK1R+ fibers in lesioned skin in patients with AD have been reported [141]. However, a recent study revealed no significant relationship between serum SP levels and AD [142]. SP is an important neuroinflammatory mediator that can modulate various cytokines by activating a wide range of immune cells, such as T cells, DCs, and eosinophils [139]. SP can stimulate MC degranulation and release histamine, leukotriene B4, prostaglandin D2, tumor necrosis factor α (TNF-α), vascular endothelial growth factor (VEGF), and IL-1 [143]. In addition to NK1R, SP can also activate MCs via MRGPCR [144]. Moreover, SP and IL-33 can stimulate IL-1β and TNF-α from MCs [145,146]. SP and CGRP can induce the release of proinflammatory cytokines IL-1β, IL-6, IL-10, NGF, TNF-α, and IL-10 from keratinocytes with an autocrine effect [147]. It has been shown that injection of SP may induce itch by NK1R [148]. However, another group showed that SP activated MRGPCR on sensory neurons to induce itching in mice [149,150]. NK1R is expressed in the DRG, spinal horns, and brain areas responsible for itch signaling [151,152,153], and a recent study demonstrated that spinal NK1R neurons mediate itching in mice [152]. In line with the findings above, NK1R may have a central role in potentiating pruritus from the periphery to the spinothalamic tract and then to the brain cortex. It showed that aprepitant, a high-affinity NK-1 receptor antagonist, significantly inhibited the scratching behavior in an NC/Nga AD mouse model [154]. Aprepitant was effective for treating chronic pruritus [155]; however, no additive effect of aprepitant was observed in a study conducted in patients with moderate to severe AD who were treated with TCS [156]. Tradipitant, an NK1 receptor antagonist, demonstrated reduction of pruritus and improvement of sleep in mild atopic eczema in a phase 3 clinical trial (EPIONE trial) [19]. Another phase 3 clinical trial (EPIONE2 trial) is currently recruiting. Serlopitant, an NK-1 receptor antagonist, failed to meet the primary endpoint of pruritus reduction in a clinical phase 2 trial.

Calcitonin gene-related peptide (CGRP) is a 37-amino-acid peptide that is present in primary afferent sensory fibers. There are two types of human CGRP, CGRP-α and CGRP-β, of which CGRP-α is considered the principal form found in the central and peripheral nervous systems. In addition to cutaneous nerve endings, CGRP can be produced by other cell types, such as LCs, monocytes, and keratinocytes [157]. CGRP can activate MC degranulation and act on keratinocytes to produce inflammatory cytokines [147,157]. Importantly, CGRP biases LCs towards a Th2 pole, which facilitates atopic skin diathesis [158]. Increased serum CGRP levels and lesioned CGRP+ nerve fibers have been reported in patients with AD [159,160]. A study by McCoy et al. reported that ablation of CGRPα-expressing sensory neurons reduced sensitivity to capsaicin, heat, and itch, indicating that CGRP-positive sensory neurons are required for the perception of an itch [161]. Hypersecretion of SP accompanied by hyposecretion of CGRP in the skin was reported in an AD mouse model [162]. In contrast, Antoh et al. reported increased expression of SP+ and CGRP+ unmyelinated C fibers, which may involve the induction of pruritus in a dry skin mouse model [163]. This discrepancy indicates that other immune responses may be indirectly affected by these neuropeptides. The high co-localization of ETAR, ET-1, and CGRP in DRG has been demonstrated in a study by Kido-Nakahara et al., indicating the possible modulatory function of CGRP with other known pruritogens [120].

Apart from SP and CGRP, other neuropeptides are also involved in AD for pruritus transmission and inflammation. Increased serum VIP levels are correlated with pruritus [164,165] and enhanced serum neuropeptide Y (NPY) levels are observed in patients with AD [160]. VIP promotes Th2 cell survival and differentiation while inhibiting Th1 cell differentiation [166]. NPY enhances IL-4 production, promotes Th2 differentiation, and is required for type 2 responses [167]. Enhanced expression in the skin and serum levels of GRP and correlation with pruritus and severity in patients with AD have been reported [168,169]. Lou et al. demonstrated that IL-22 induced expression of GRP receptors (GRPR) in keratinocytes and dermal GRP+ immune cells in the skin of patients with AD, of which the dermal GRP+ cells were correlated with AD severity and degree of pruritus [168]. Significant amounts of sensory neurons express SP or GRP [151]. Additionally, GRP is a crucial spinal neurotransmitter for itch [170], and the latest study showed that spinal dorsal horn NK1R neurons, which are responsible for spinal pruritus, contained GRPR [152], and upregulation of serum neurotensin levels and gene expression in the lesioned skin of patients with AD. The possible interaction between neurotensin and MCs may contribute to the pathogenesis of AD [171]. Different levels of somatostatin receptor expression have been demonstrated in the skin of patients with AD but their pathogenic role is unclear [172].

### 3.10. Toll-Like Receptors (TLRs)

TLRs are cellular sensors that initiate immune responses to noxious stimuli. Accordingly, they act as an interface between innate and adaptive immunity [173]. TLR2 and TLR4 polymorphisms are associated with high susceptibility to AD [174]. Furthermore, the excessive expression of chemokine mRNA by TLR2 activation may contribute to the development of AD [175]. Impaired TLR2 signaling with defective Th1 and Th17/22 cytokine expression from the PBMCs of patients with AD may skew towards Th2 response [176]. Elevated stratum corneum expression of TLR3 has been shown, which is closely associated with lesion severity and skin hydration in patients with AD [177]. Another group also showed increased TLR3 expression in chronic skin lesions of patients with AD [178]. Moreover, ET-1, TSLP, and IL-33, the pruritogens reviewed above, can also be induced by TLR3 activation in keratinocytes [56,178,179]. Notably, TLR3, TLR4, and TLR7 are located in the sensory neurons if the DRG. TLR3 and TLR7 colocalize with TRPV1 channels and GRP neurons, the itch signaling components, and their ligands can activate DRG neurons [180,181]. TLR3 knockdown in DRG showed attenuation of pruritus in a dry skin mouse model [180]. TLR4 is also co-expressed with TRPV1 channels in DRG sensory neurons, and a recent study showed that TLR4 promotes CGRP release from afferent neurons [182,183]. It has been reported that TLR7 was responsible for non-histamine itch [181]; however, a TLR7 agonist, imiquimod, showed a TRPV1-dependent but TLR7-independent itch pathway [184]. Further studies are required to understand the mechanism of TLR in pruritus.

## 4. TCS and Topical Calcineurin Inhibitors (TCI) in Pruritus Control

Targeting specific pruritogens or inflammatory cytokines reviewed above provides accurate options for the treatment of AD. Traditional treatments of TCS and TCI are effective in reducing pruritus, especially for mild AD [185]. The anti-inflammatory effect of topical steroids and TCI may indirectly decrease the degree of pruritus in AD [186]. Additionally, TCI also suppress nerve fiber activation, desensitize TRPV1 channels, and inhibit the expression of the pruritogens CGRP, SP, IL-33, and IL-31 [75,187,188]. Prolonged external application of corticosteroids may cause adverse effects such as cutaneous atrophy and ecchymosis, while common side effects of TCI use include pruritus, stinging, and burning at the application site [185].

## 5. MCs as a Therapeutic Target in AD and AD-Related Pruritus

Given that MCs are a huge source of pruritogens and active inflammatory mediators, targeting MCs is theoretically feasible. Ketotifen, an H1 antihistamine and MC stabilizer, is known to relieve some of the allergic symptoms in AD. However, other MC stabilizers have limited efficacy in AD, such as sodium cromoglycate and nedocromil sodium [189,190]. New agents targeting the reduction of MCs have potential roles in the treatment of AD [191]. ABT-737, an inhibitor of Bcl-2, induces apoptosis in MCs and decreases the levels of AD-related biomarkers, including IgE, histamine, TSLP, and inflammatory cytokines in vitro, reducing AD-like clinical symptoms in an AD mouse model [192]. Imatinib, an inhibitor targeting c-kit and tyrosine kinases, can reduce the number of MCs and is a treatment for systemic mastocytosis [193]. Further evaluation of agents targeting MC activation and MC mediators is necessary.

## 6. Treatment Strategy for AD Patients with Concomitant Chronic Renal Insufficiency or Chronic Cholestatic Diseases

The prevalence of chronic renal insufficiency associated with pruritus (uremic pruritus) is high, affecting up to half of the patients undergoing dialysis [194]. The pathophysiology of uremic pruritus is unclear and multifactorial [195]. Therefore, multiple therapies have been used, including gabapentin, pregabalin, MC stabilizers, phototherapy, hemodialysis modifications, and multiple other systemic and topical treatments. Among them, the most effective treatment, with a large body of evidence, is gabapentin [196]. As there is no acute skin inflammation in uremic pruritus, the role of altered immune signaling in pruritogenic nerve activation and central sensitization has been elucidated. The potential role of dupilumab in the signaling of IL-4 and/or IL-13 on sensory neurons in the management of uremic pruritus has been demonstrated [197]. IL-31 may also be a future therapeutic target, because increased serum IL-31 levels have been observed in patients with uremic pruritus [198]. Similarly, the pathophysiology of cholestatic pruritus is also multifactorial, and bile acid, lysophosphatidic acid, and bilirubin are potentially important mediators of cholestatic itch. Treatments include cholestyramine, rifampin, opioid antagonists, sertraline, a selective serotonin reuptake inhibitor, and dronabinol. For refractory cases, further interventions are required, including phototherapy, albumin dialysis, plasmapheresis, and nasobiliary drainage [199]. Of note, general preventive measures, including aggressive skin hydration and patient education, are critical for preventing additional complications from scratching.

## 7. Conclusions

Taken together, these results indicate that pruritogen is one of the key players in the pathogenesis of AD. We can no longer only focus on pruritogen as a substance that induces desire to scratch, as the exogenous and endogenous pruritogens also initiate the inflammation cascade in AD and act as a bridge between the skin, immune system, and nervous system. In turn, inflammatory mediators evoke itch directly via the corresponding receptors on cutaneous free nerve endings. The neuroimmune axis provides new insight into the molecular mechanisms of epithelial cells, immune cells and nervous system communication.

Currently, great efforts on the pathophysiology of pruritus have provided detailed information about the neurocutaneous interaction in chronic pruritic dermatosis. This helps to develop targeted therapies not only in AD, but also in other recalcitrant itch disorders such as psoriasis and prurigo nodularis. Biologics targeting IL-4, IL-13, IL-31, and others have been developed and have shown optimistic results. This review summarizes the direction of future therapeutics, focusing on the current ongoing and experimental drugs based on potential biological targets. Due to the complexity of the inflammatory process and itch transmission in AD, treatment remains challenging and multidirectional treatments are necessary. Further investigation is warranted to develop better treatment options for recalcitrant pruritic dermatosis.

## Figures and Tables

**Table 1:** Potential experimental targeted biologics against pruritogens in AD.

	Target	Study Phase (Trial Identification)	Enrollment	Efficacy	Status	Ref.
JNJ 39758979	H4R	Phase 2a (NCT01497119)	*n* = 88	Improvement of histamine-related pruritus. Improvement of Pruritus in moderate AD.	Termination due to agranulocytosis	[9]
Adriforant (ZPL-3893787)	H4R	Phase 2 (NCT02424253)	*n* = 98	At week 8, Decreased EASI score 50% vs. 27%. Decreased SCORAD score 40% vs 26%. IGA 0/1 18.5% vs. 9.1%.	Completed	[10]
Rupatatine	H1R and PAF	Phase 3 (JapicCTI-152787)	*n* = 66	Improvement of total pruritus score	Completed	[11]
Tezepelumab (plus TCS)	TSLP	Phase 2a (NCT02525094)	*n* = 113	At week 12, Pruritus NRS 31.53 vs 21.05. At week 16, IGA response rate 29.4% vs. 12.9%.	Completed	[12]
MK8226	TSLP receptor inhibitor	Phase 1b (NCT01732510)	*n* = 65	Results not yet released	Terminate due to business reason	
Etokimab (ANB020)	IL-33	Phase 2a (NCT03533751)	*n* = 12	At day 29, EASI 50 83.3%. EASI 75 33%. At day 57, EASI 50 75%. EASI 75 42%. Significant improvement of DLQI and 5D itch scales.	Completed	[13]
Etokimab (ANB020)	IL-33	Phase 2b ATLAS trail	*n* = 300	Results not yet released	Recruiting	
REGN3500	IL-33	Phase 2 (NCT03736967)	*n* = 206	Results not yet released	Completed	
PF-06817024	IL-33	Phase 1 (NCT02743871)	*n* = 98	Results not yet released	Completed	
Pitrakinra (AER 100, BAY 16-9996)	IL-4 alpha receptor	Phase 2a (NCT00676884)	*n* = 25	Results not yet released	Completed	
CM310	IL-4 alpha receptor	Phase 2b (NCT04805411)	*n* = 120	Recruiting	Recruiting	
CBP-201	IL-4 alpha receptor	Phase 2 (NCT04444752)	*n* = 220	Active, not recruiting	Active, not recruiting	
AK210	IL-4 alpha receptor	Phase 1 (NCT04256174)	*n* = 70	Recruiting	Recruiting	
Tralokinumab	IL-13	Phase 3 ECZTRA 1 trial (NCT03131648)	*n* = 802	At week 16 IGA 0/1 15.8% vs. 7.1%. EASI 75 25% vs. 12.7. NRS > 4 points improvement 20% vs. 10.9%	Completed	[14]
Tralokinumab	IL-13	Phase 3 ECZTRA 2 trial (NCT03160885)	*n* = 794	At week16 IGA 0/1 22.2% vs. 10.9%. EASI 75 33.2% vs. 11.4%. Pruritus NRS > 4 points improvement 25% vs. 9.5%.	Completed	[14]
Tralokinumab (plus TCS)	IL-13	Phase 3 ECZTRA 3 trial(NCT03363854)	*n* = 380	At week 16 IGA 0/1 38.9% vs. 26.2%. EASI 75 56% vs. 35.7%. Pruritus NRS > 4 points improvement 45.4% vs. 34.1%.	Completed	[15]
Lebrikizumab	IL-13	Phase 2b (NCT03443024)	*n* = 280	At week 16 EASI improvement 72.1% (250 mg q2w) vs. 69.2% (250 mg q4w) vs. 62.3% (125 mg q4w) vs. 41.1% (placebo). Pruritus NRS > 4 points improvement 70% (250mg q2w) vs. 27.5% (placebo).	Completed	[16]
Nemolizumab (plus TCS)	IL-31 receptor alpha inhibitor	Phase 2b (NCT03100344)	*n* = 226	At week 24 EASI improvement 68.8% vs. 51.2%. PP-NRS reduction 68.6% vs. 34.3%	Completed	[17]
Nemolizumab (plus TCS)	IL-31 receptor alpha inhibitor	Phase 3 (JapicCTI number, 173740)	*n* = 270	VAS score for pruritus improvement 42.8% vs. 21.4%	Completed	[18]
BMS-981164	IL-31	Phase 1 (NCT01614756)	*n* = 93	Results not yet released	Completed	
Tradipitant	Neurokinin receptor	Phase 3 EPIONE trial (NCT03568331)	*n* = 375	Improvement of pruritus and sleep in mild lesion AD	Completed	[19]
Tradipitant	Neurokinin receptor	Phase 3 EPIONE2 trial (NCT04140695)	*n* = 200	Recruiting	Recruiting	
Serlopitant	Neurokinin receptor	Phase 2 (NCT02975206)	*n* = 484	Failed to meet the primary endpoint WI-NRS score	Completed	

AD, atopic dermatitis; EASI, eczema area and severity index; EASI 50, improvement of greater than or equal to 50% in EASI; EASI 75, improvement of greater than or equal to 75% in EASI; NRS, numeric rating scale; SCORA, scoring of atopic dermatitis; IGA, investigators global assessment; IGA 0/1, IGA 0 or 1 (clear or almost clear) plus greater than or equal to 2 grade improvement; PP-NRS, peak pruritus numeric rating scales; TCS, topical corticosteroids; WI-NRS, worst itch numeric rating scale. The data above was retrieved by 5 June 2021.

## References

[1] Wahlgren C.F. (1999). Itch and atopic dermatitis: An overview. J. Derm..

[2] Weidinger S., Beck L.A., Bieber T., Kabashima K., Irvine A.D. (2018). Atopic dermatitis. Nat. Rev. Dis. Primers.

[3] Kabashima K. (2013). New concept of the pathogenesis of atopic dermatitis: Interplay among the barrier, allergy, and pruritus as a trinity. J. Derm. Sci..

[4] Han L., Dong X. (2014). Itch mechanisms and circuits. Annu. Rev. Biophys..

[5] Kabata H., Artis D. (2019). Neuro-immune crosstalk and allergic inflammation. J. Clin. Investig..

[6] Simons F.E., Simons K.J. (2011). Histamine and H1-antihistamines: Celebrating a century of progress. J. Allergy Clin. Immunol..

[7] Oetjen L.K., Mack M.R., Feng J., Whelan T.M., Niu H., Guo C.J., Chen S., Trier A.M., Xu A.Z., Tripathi S.V. (2017). Sensory neurons co-opt classical immune signaling pathways to mediate chronic itch. Cell.

[8] Wilson S.R., The L., Batia L.M., Beattie K., Katibah G.E., McClain S.P., Pellegrino M., Estandian D.M., Bautista D.M. (2013). The epithelial cell-derived atopic dermatitis cytokine TSLP activates neurons to induce itch. Cell.

[9] Murata Y., Song M., Kikuchi H., Hisamichi K., Xu X.L., Greenspan A., Kato M., Chiou C.F., Kato T., Guzzo C. (2015). Phase 2a, randomized, double-blind, placebo-controlled, multicenter, parallel-group study of a H4R-antagonist (JNJ-39758979) in Japanese adults with moderate atopic dermatitis. J. Derm..

[10] Werfel T., Layton G., Yeadon M., Whitlock L., Osterloh I., Jimenez P., Liu W., Lynch V., Asher A., Tsianakas A. (2019). Efficacy and safety of the histamine H4 receptor antagonist ZPL-3893787 in patients with atopic dermatitis. J. Allergy Clin. Immunol..

[11] Hide M., Suzuki T., Tanaka A., Aoki H. (2019). Long-term safety and efficacy of rupatadine in Japanese patients with itching due to chronic spontaneous urticaria, dermatitis, or pruritus: A 12-month, multicenter, open-label clinical trial. J. Derm. Sci..

[12] Simpson E.L., Parnes J.R., She D., Crouch S., Rees W., Mo M., Merwe R.V.D. (2019). Tezepelumab, an anti-thymic stromal lymphopoietin monoclonal antibody, in the treatment of moderate to severe atopic dermatitis: A randomized phase 2a clinical trial. J. Am. Acad. Derm..

[13] Chen Y.L., Gutowska-Owsiak D., Hardman C.S., Westmoreland M., MacKenzie T., Cifuentes L., Waithe D., Lloyd-Lavery A., Marquette A., Londei M. (2019). Proof-of-concept clinical trial of Etokimab shows a key role for IL-33 in atopic dermatitis pathogenesis. Sci. Transl. Med..

[14] Wollenberg A., Blauvelt A., Guttman-Yassky E., Worm M., Lynde C., Lacour J.P., Spelman L., Katoh N., Saeki H., Poulin Y. (2020). Tralokinumab for moderate-to-severe atopic dermatitis: Results from two 52-week, randomized, double-blind, multicentre, placebo-controlled phase III trials (ECZTRA 1 and ECZTRA 2). Br. J. Derm..

[15] Silverberg J.I., Toth D., Bieber T., Alexis A.F., Elewski B.E., Pink A.E., Hijnen D., Jensen T.N., Bang B., Olsen C.K. (2021). Tralokinumab plus topical corticosteroids for the treatment of moderate-to-severe atopic dermatitis: Results from the double-blind, randomized, multicentre, placebo-controlled phase III ECZTRA 3 trial. Br. J. Derm..

[16] Guttman-Yassky E., Blauvelt A., Eichenfield L.F., Paller A.S., Armstrong A.W., Drew J., Gopalan R., Simpson E.L. (2020). Efficacy and safety of Lebrikizumab, a high-affinity interleukin 13 inhibitor, in adults with moderate to severe atopic dermatitis. JAMA Derm..

[17] Silverberg J.I., Pinter A., Pulka G., Poulin Y., Bouaziz J.-D., Wollenberg A., Murrell D.F., Alexis A., Lindsey L., Ahmad F. (2020). Phase 2b randomized study of nemolizumab in adults with moderate-to-severe atopic dermatitis and severe pruritus. J. Allergy Clin. Immunol..

[18] Kabashima K., Matsumura T., Komazaki H., Kawashima M. (2020). Trial of nemolizumab and topical agents for atopic dermatitis with pruritus. N. Engl. J. Med..

[19] Welsh S.E., Xiao C., Kaden A.R., Brzezynski J.L., Mohrman M.A., Wang J., Smieszek S.P., Przychodzen B., Ständer S., Polymeropoulos C. (2020). Neurokinin-1 receptor antagonist tradipitant has mixed effects on itch in atopic dermatitis: Results from EPIONE, a randomized clinical trial. J. Eur. Acad. Derm. Venereol..

[20] Rossbach K., Nassenstein C., Gschwandtner M., Schnell D., Sander K., Seifert R., Stark H., Kietzmann M., Bäumer W. (2011). Histamine H1, H3 and H4 receptors are involved in pruritus. Neuroscience.

[21] Ohtsu H., Seike M. (2016). Histamine and histamine receptors in allergic dermatitis. Handb. Exp. Pharm..

[22] Takahashi A., Tani S., Murota H., Katayama I. (2016). Histamine modulates sweating and affects clinical manifestations of atopic dermatitis. Curr. Probl. Derm..

[23] Gutzmer R., Mommert S., Gschwandtner M., Zwingmann K., Stark H., Werfel T. (2009). The histamine H4 receptor is functionally expressed on Th2 cells. J. Allergy Clin. Immunol..

[24] Glatzer F., Gschwandtner M., Ehling S., Rossbach K., Janik K., Klos A., Bäumer W., Kietzmann M., Werfel T., Gutzmer R. (2013). Histamine induces proliferation in keratinocytes from patients with atopic dermatitis through the histamine 4 receptor. J. Allergy Clin. Immunol..

[25] Connelly W.M., Shenton F.C., Lethbridge N., Leurs R., Waldvogel H.J., Faull R.L.M., Lees G., Chazot P.L. (2009). The histamine H4 receptor is functionally expressed on neurons in the mammalian cns. Br. J. Pharm..

[26] Rossbach K., Schaper K., Kloth C., Gutzmer R., Werfel T., Kietzmann M., Bäumer W. (2016). Histamine H4 receptor knockout mice display reduced inflammation in a chronic model of atopic dermatitis. Allergy.

[27] Schaper K., Rossbach K., Köther B., Stark H., Kietzmann M., Werfel T., Gutzmer R. (2016). Stimulation of the histamine 4 receptor upregulates thymic stromal lymphopoietin (TSLP) in human and murine keratinocytes. Pharm. Res..

[28] Mommert S., Hüer M., Schaper-Gerhardt K., Gutzmer R., Werfel T. (2020). Histamine up-regulates oncostatin M expression in human M1 macrophages. Br. J. Pharm..

[29] Cowden J.M., Zhang M., Dunford P.J., Thurmond R.L. (2010). The histamine H4 receptor mediates inflammation and pruritus in Th2-dependent dermal inflammation. J. Investig. Derm..

[30] Köchling H., Schaper K., Wilzopolski J., Gutzmer R., Werfel T., Bäumer W., Kietzmann M.R., Rossbach K. (2017). Combined treatment with H1 and H4 receptor antagonists reduces inflammation in a mouse model of atopic dermatitis. J. Derm. Sci..

[31] Otsuka A., Honda T., Doi H., Miyachi Y., Kabashima K. (2011). An H1-histamine receptor antagonist decreases serum Interleukin-31 levels in patients with atopic dermatitis. Br. J. Derm..

[32] Kollmeier A., Franke K., Chen B., Dunford P.J., Greespan A.J., Xia Y., Xu X.L., Zhou B., Thurmond R.L. (2014). The histamine H4 receptor antagonist, JNH 39758979, is effective in reducing histamine-induced pruritus in a randomized clinical study in healthy subjects. J. Pharm. Exp..

[33] Schauberger E., Peinhaupt M., Cazares T., Lindsley A.W. (2016). Lipid mediators of allergic disease: Pathways, treatments, and emerging therapeutic targets. Curr. Allergy Asthma Rep..

[34] Dyer K.D., Percopo C.M., Xie Z., Yang Z., Kim J.D., Davoine F., Lacy P., Druey K.M., Moqbel R., Rosenberg H.F. (2010). Mouse and human eosinophils degranulate in response to platelet-activating factor (PAF) and lysoPAF via a PAF-receptor–independent mechanism: Evidence for a novel receptor. J. Immunol..

[35] Petersen L.J., Church M.K., Skov P.S. (1997). Platelet-activating factor induces histamine release from human skin mast cells in vivo, which is reduced by local nerve blockade. J. Allergy Clin. Immunol..

[36] Lee C.-H. (2010). Progress of pruritus research in atopic dermatitis. Biomol. Ther..

[37] Ocana J.A., Romer E., Sahu R., Pawelzik S.-C., FitzGerald G.A., Kaplan M.H., Travers J.B. (2018). Platelet-activating factor–induced reduction in contact hypersensitivity responses is mediated by mast cells via cyclooxygenase-2–dependent mechanisms. J. Immunol..

[38] Lee S.E., Jeong S.K., Lee S.H. (2010). Protease and protease-activated receptor-2 signaling in the pathogenesis of atopic dermatitis. Yonsei Med. J..

[39] Moniaga C.S., Jeong S.K., Egawa G., Nakajima S., Hara-Chikuma M., Jeon J.E., Lee S.H., Hibino T., Miyachi Y., Kabashima K. (2013). Protease activity enhances production of thymic stromal lymphopoietin and basophil accumulation in flaky tail mice. Am. J. Pathol..

[40] Ramachandran R., Hollenberg M.D. (2008). Proteinases and signalling: Pathophysiological and therapeutic implications via PARs and more. Br. J. Pharm..

[41] Nomura H., Suganuma M., Takeichi T., Kono M., Isokane Y., Sunagawa K., Kobashi M., Sugihara S., Kajita A., Miyake T. (2020). Multifaceted analyses of epidermal serine protease activity in patients with atopic dermatitis. Int. J. Mol. Sci..

[42] Steinhoff M., Neisius U., Ikoma A., Fartasch M., Heyer G., Skov P.S., Luger T.A., Schmelz M. (2003). Proteinase-activated receptor-2 mediates itch: A novel pathway for pruritus in human skin. J. Neurosci..

[43] Briot A., Deraison C., Lacroix M., Bonnart C., Robin A., Besson C., Dubus P., Hovnanian A. (2009). Kallikrein 5 induces atopic dermatitis–like lesions through PAR2-mediated thymic stromal lymphopoietin expression in netherton syndrome. J. Exp. Med..

[44] Guo C.J., Mack M.R., Oetjen L.K., Trier A.M., Council M.L., Pavel A.B., Guttman-Yassky E., Kim B.S., Liu Q. (2020). Kallikrein 7 promotes atopic dermatitis-associated itch independently of skin inflammation. J. Investig. Derm..

[45] Zhu Y., Underwood J., Macmillan D., Shariff L., O’Shaughnessy R., Harper J.I., Pickard C., Friedmann P.S., Healy E., Di W.-L. (2017). Persistent kallikrein 5 activation induces atopic dermatitis-like skin architecture independent of PAR2 activity. J. Allergy Clin. Immunol..

[46] Zhao J., Munanairi A., Liu X.-Y., Zhang J., Hu L., Hu M., Bu D., Liu L., Xie Z., Kim B.S. (2020). PAR2 mediates itch via TRPV3 signaling in keratinocytes. J. Investig. Derm..

[47] Buhl T., Ikoma A., Kempkes C., Cevikbas F., Sulk M., Buddenkotte J., Akiyama T., Crumrine D., Camerer E., Carstens E. (2020). Protease-activated receptor-2 regulates neuro-epidermal communication in atopic dermatitis. Front. Immunol..

[48] Braz J.M., Dembo T., Charruyer A., Ghadially R., Fassett M.S., Basbaum A.I. (2021). Genetic priming of sensory neurons in mice that overexpress PAR2 enhances allergen responsiveness. Proc. Natl. Acad. Sci. USA.

[49] Akiyama T., Carstens M.I., Carstens E. (2009). Excitation of mouse superficial dorsal horn neurons by histamine and/or PAR-2 agonist: Potential role in itch. J. Neurophysiol..

[50] Amadesi S., Nie J., Vergnolle N., Cottrell G.S., Grady E.F., Trevisani M., Manni C., Geppetti P., McRoberts J.A., Ennes H. (2004). Protease-activated receptor 2 sensitizes the capsaicin receptor transient receptor potential vanilloid receptor 1 to induce hyperalgesia. J. Neurosci..

[51] Chung K., Pitcher T., Grant A.D., Hewitt E., Lindstrom E., Malcangio M. (2019). Cathepsin S acts via protease-activated receptor 2 to activate sensory neurons and induce itch-like behaviour. Neurobiol. Pain..

[52] Barr T.P., Garzia C., Guha S., Fletcher E.K., Nguyen N., Wieschhaus A.J., Ferrer L., Covic L., Kuliopulos A. (2019). PAR2 pepducin-based suppression of inflammation and itch in atopic dermatitis models. J. Investig. Derm..

[53] Andoh T., Kuraishi Y. (2014). Antipruritic mechanisms of topical E6005, a phosphodiesterase 4 inhibitor: Inhibition of responses to proteinase-activated receptor 2 stimulation mediated by increase in intracellular cyclic AMP. J. Derm. Sci..

[54] Varricchi G., Pecoraro A., Marone G., Criscuolo G., Spadaro G., Genovese A., Marone G. (2018). Thymic stromal lymphopoietin isoforms, inflammatory disorders, and cancer. Front. Immunol..

[55] Harada M., Hirota T., Jodo A.I., Doi S., Kameda M., Fujita K., Miyatake A., Enomoto T., Noguchi E., Yoshihara S. (2009). Functional analysis of the thymic stromal lymphopoietin variants in human bronchial epithelial cells. Am. J. Respir. Cell Mol. Biol..

[56] Xie Y., Takai T., Chen X., Okumura K., Ogawa H. (2012). Long TSLP transcript expression and release of TSLP induced by TLR ligands and cytokines in human keratinocytes. J. Derm. Sci..

[57] Dong H., Hu Y., Liu L., Zou M., Huang C., Luo L., Yu C., Wan X., Zhao H., Chen J. (2016). Distinct roles of short and long thymic stromal lymphopoietin isoforms in house dust mite-induced asthmatic airway epithelial barrier disruption. Sci. Rep..

[58] Bjerkan L., Sonesson A., Schenck K. (2016). Multiple functions of the new cytokine-based antimicrobial peptide thymic stromal lymphopoietin (TSLP). Pharmaceuticals.

[59] Lee E.B., Kim K.W., Hong J.Y., Jee H.M., Sohn M.H., Kim K.E. (2010). Increased serum thymic stromal lymphopoietin in children with atopic dermatitis. Pediatr. Allergy Immunol..

[60] Takai T. (2012). TSLP expression: Cellular sources, triggers, and regulatory mechanisms. Allergol. Int..

[61] Watanabe N., Hanabuchi S., Soumelis V., Yuan W., Ho S., de Waal Malefyt R., Liu Y.-J. (2004). Human thymic stromal lymphopoietin promotes dendritic cell–mediated CD4+ T cell homeostatic expansion. Nat. Immunol..

[62] Pattarini L., Trichot C., Bogiatzi S., Grandclaudon M., Meller S., Keuylian Z., Durand M., Volpe E., Madonna S., Cavani A. (2017). TSLP-activated dendritic cells induce human T follicular helper cell differentiation through OX40-ligand. J. Exp. Med..

[63] Marschall P., Wei R., Segaud J., Yao W., Hener P., German B.F., Meyer P., Hugel C., Ada Da Silva G., Braun R. (2021). Dual function of Langerhans cells in skin TSLP-promoted TFH differentiation in mouse atopic dermatitis. J. Allergy Clin. Immunol..

[64] Tatsuno K., Fujiyama T., Yamaguchi H., Waki M., Tokura Y. (2015). TSLP directly interacts with skin-homing Th2 cells highly expressing its receptor to enhance IL-4 production in atopic dermatitis. J. Investig. Derm..

[65] Rochman Y., Dienger-Stambaugh K., Richgels P.K., Lewkowich I.P., Kartashov A.V., Barski A., Khurana Hershey G.K., Leonard W.J., Singh H. (2018). TSLP signaling in CD4+T cells programs a pathogenic T helper 2 cell state. Sci. Signal..

[66] Ochiai S., Jagot F., Kyle R.L., Hyde E., White R.F., Prout M., Schmidt A.J., Yamane H., Lamiable O., Le Gros G. (2018). Thymic stromal lymphopoietin drives the development of IL-13+ Th2 cells. Proc. Natl. Acad. Sci. USA.

[67] Wallmeyer L., Dietert K., Sochorová M., Gruber A.D., Kleuser B., Vávrová K., Hedtrich S. (2017). TSLP is a direct trigger for T cell migration in filaggrin-deficient skin equivalents. Sci. Rep..

[68] Salter B.M., Oliveria J.P., Nusca G., Smith S.G., Watson R.M., Comeau M., Sehmi R., Gauvreau G.M. (2015). Thymic stromal lymphopoietin activation of basophils in patients with allergic asthma is IL-3 dependent. J. Allergy Clin. Immunol..

[69] Salabert-Le Guen N., Hémont C., Delbove A., Poli C., Braudeau C., Fantou A., Amouriaux K., Bériou G., Martin J.C., Colas L. (2018). Thymic stromal lymphopoietin does not activate human basophils. J. Allergy Clin. Immunol..

[70] Kim J.H., Bae H.C., Ko N.Y., Lee S.H., Jeong S.H., Lee H., Ryu W.-I., Kye Y.C., Son S.W. (2015). Thymic stromal lymphopoietin downregulates filaggrin expression by signal transducer and activator of transcription 3 (STAT3) and extracellular signal-regulated kinase (ERK) phosphorylation in keratinocytes. J. Allergy Clin. Immunol..

[71] Leyva-Castillo J.M., Hener P., Jiang H., Li M. (2013). TSLP produced by keratinocytes promotes allergen sensitization through skin and thereby triggers atopic march in mice. J. Investig. Derm..

[72] Unutmaz D., Moussion C., Ortega N., Girard J.-P. (2008). The IL-1-like cytokine IL-33 is constitutively expressed in the nucleus of endothelial cells and epithelial cells in vivo: A novel ‘alarmin’?. PLoS ONE.

[73] Imai Y. (2019). Interleukin-33 in atopic dermatitis. J. Derm. Sci..

[74] Martin N.T., Martin M.U. (2016). Interleukin 33 is a guardian of barriers and a local alarmin. Nat. Immunol..

[75] Savinko T., Matikainen S., Saarialho-Kere U., Lehto M., Wang G., Lehtimäki S., Karisola P., Reunala T., Wolff H., Lauerma A. (2012). IL-33 and ST2 in atopic dermatitis: Expression profiles and modulation by triggering factors. J. Investig. Derm..

[76] Kindi A.A., Williams H., Matsuda K., Alkahtani A.M., Saville C., Bennett H., Alshammari Y., Tan S.Y., O’Neill C., Tanaka A. (2021). Staphylococcus aureus second immunoglobulin-binding protein drives atopic dermatitis via IL-33. J. Allergy Clin. Immunol..

[77] Tamagawa-Mineoka R., Okuzawa Y., Masuda K., Katoh N. (2014). Increased serum levels of interleukin 33 in patients with atopic dermatitis. J. Am. Acad. Derm..

[78] Nakamura N., Tamagawa-Mineoka R., Yasuike R., Masuda K., Matsunaka H., Murakami Y., Yokosawa E., Katoh N. (2019). Stratum corneum interleukin-33 expressions correlate with the degree of lichenification and pruritus in atopic dermatitis lesions. Clin. Immunol..

[79] Franke K., Wang Z., Zuberbier T., Babina M. (2021). Cytokines stimulated by IL-33 in human skin mast cells: Involvement of NF-κB and p38 at distinct levels and potent co-operation with FcεRI and MRGPRX2. Int. J. Mol. Sci..

[80] Imai Y., Yasuda K., Nagai M., Kusakabe M., Kubo M., Nakanishi K., Yamanishi K. (2019). IL-33–induced atopic dermatitis–like inflammation in mice is mediated by group 2 innate lymphoid cells in concert with basophils. J. Investig. Derm..

[81] Murakami-Satsutani N., Ito T., Nakanishi T., Inagaki N., Tanaka A., Vien P.T.X., Kibata K., Inaba M., Nomura S. (2014). IL-33 promotes the induction and maintenance of Th2 immune responses by enhancing the function of OX40 ligand. Allergol. Int..

[82] Ryu W.-I., Lee H., Bae H.C., Ryu H.J., Son S.W. (2016). IL-33 down-regulates filaggrin expression by inducing STAT3 and ERK phosphorylation in human keratinocytes. J. Derm. Sci..

[83] Liu B., Tai Y., Achanta S., Kaelberer M.M., Caceres A.I., Shao X., Fang J., Jordt S.-E. (2016). IL-33/ST2 signaling excites sensory neurons and mediates itch response in a mouse model of poison ivy contact allergy. Proc. Natl. Acad. Sci. USA.

[84] Du L., Hu X., Yang W., Yasheng H., Liu S., Zhang W., Zhou Y., Cui W., Zhu J., Qiao Z. (2019). Spinal IL-33/ST2 signaling mediates chronic itch in mice through the astrocytic JAK2-STAT3 cascade. Glia.

[85] Howell M.D., Kim B.E., Gao P., Grant A.V., Boguniewicz M., DeBenedetto A., Schneider L., Beck L.A., Barnes K.C., Leung D.Y. (2009). Cytokine modulation of atopic dermatitis filaggrin skin expression. J. Allergy Clin. Immunol..

[86] McCormick S.M., Heller N.M. (2015). Commentary: IL-4 and IL-13 receptors and signaling. Cytokine.

[87] Chiricozzi A., Maurelli M., Peris K., Girolomoni G. (2020). Targeting IL-4 for the treatment of atopic dermatitis. Immunotargets Ther..

[88] Bao K., Reinhardt R.L. (2015). The differential expression of IL-4 and IL-13 and its impact on type-2 immunity. Cytokine.

[89] Furue K., Ito T., Tsuji G., Ulzii D., Vu Y.H., Kido-Nakahara M., Nakahara T., Furue M. (2019). The IL-13-OVOL1–FLG axis in atopic dermatitis. Immunology.

[90] Miake S., Tsuji G., Takemura M., Hashimoto-Hachiya A., Vu Y.H., Furue M., Nakahara T. (2019). IL-4 augments IL-31/IL-31 receptor alpha interaction leading to enhanced CCL 17 and CCL 22 production in dendritic cells: Implications for atopic dermatitis. Int. J. Mol. Sci..

[91] Zheng T., Oh M.H., Oh S.Y., Schroeder J.T., Glick A.B., Zhu Z. (2009). Transgenic expression of interleukin-13 in the skin induces a pruritic dermatitis and skin remodeling. J. Investig. Derm..

[92] Bitton A., Avlas S., Reichman H., Itan M., Karo-Atar D., Azouz N.P., Rozenberg P., Diesendruck Y., Nahary L., Rothenberg M.E. (2020). A key role for IL-13 signaling via the type 2 IL-4 receptor in experimental atopic dermatitis. Sci. Immunol..

[93] Gooderham M.J., Hong H.C.-H., Eshtiaghi P., Papp K.A. (2018). Dupilumab: A review of its use in the treatment of atopic dermatitis. J. Am. Acad. Derm..

[94] Simpson E.L., Bieber T., Guttman-Yassky E., Beck L.A., Blauvelt A., Cork M.J., Silverberg J.I., Deleuran M., Kataoka Y., Lacour J.-P. (2016). Two phase 3 trials of dupilumab versus placebo in atopic dermatitis. N. Engl. J. Med..

[95] Nakashima C., Otsuka A., Kabashima K. (2018). Interleukin-31 and interleukin-31 receptor: New therapeutic targets for atopic dermatitis. Exp. Derm..

[96] Kasraie S., Niebuhr M., Werfel T. (2009). Interleukin (IL)-31 induces pro-inflammatory cytokines in human monocytes and macrophages following stimulation with staphylococcal exotoxins. Allergy.

[97] Stott B., Lavender P., Lehmann S., Pennino D., Durham S., Schmidt-Weber C.B. (2013). Human IL-31 is induced by IL-4 and promotes Th2-driven inflammation. J. Allergy Clin. Immunol..

[98] Dillon S.R., Sprecher C., Hammond A., Bilsborough J., Rosenfeld-Franklin M., Presnell S.R., Haugen H.S., Maurer M., Harder B., Johnston J. (2004). Interleukin 31, a cytokine produced by activated T cells, induces dermatitis in mice. Nat. Immunol..

[99] Kato A., Fujii E., Watanabe T., Takashima Y., Matsushita H., Furuhashi T., Morita A. (2014). Distribution of IL-31 and its receptor expressing cells in skin of atopic dermatitis. J. Derm. Sci.

[100] Sonkoly E., Muller A., Lauerma A.I., Pivarcsi A., Soto H., Kemeny L., Alenius H., Dieu-Nosjean M.C., Meller S., Rieker J. (2006). IL-31: A new link between T cells and pruritus in atopic skin inflammation. J. Allergy Clin. Immunol..

[101] Raap U., Wichmann K., Bruder M., Stander S., Wedi B., Kapp A., Werfel T. (2008). Correlation of IL-31 serum levels with severity of atopic dermatitis. J. Allergy Clin. Immunol..

[102] Kunsleben N., Rüdrich U., Gehring M., Novak N., Kapp A., Raap U. (2015). IL-31 induces chemotaxis, calcium mobilization, release of reactive oxygen species, and CCL26 in eosinophils, which are capable to release IL-31. J. Investig. Derm..

[103] Zimmer J., Wong C.-K., Leung K.M.-L., Qiu H.-N., Chow J.Y.-S., Choi A.O.K., Lam C.W.-K. (2012). Activation of eosinophils interacting with dermal fibroblasts by pruritogenic cytokine IL-31 and alarmin IL-33: Implications in atopic dermatitis. PLoS ONE.

[104] Raap U., Gehring M., Kleiner S., Rüdrich U., Eiz-Vesper B., Haas H., Kapp A., Gibbs B.F. (2017). Human basophils are a source of—and are differentially activated by—IL-31. Clin. Exp. Allergy.

[105] Cornelissen C., Marquardt Y., Czaja K., Wenzel J., Frank J., Lüscher-Firzlaff J., Lüscher B., Baron J.M. (2012). IL-31 regulates differentiation and filaggrin expression in human organotypic skin models. J. Allergy Clin. Immunol..

[106] Hänel K.H., Pfaff C.M., Cornelissen C., Amann P.M., Marquardt Y., Czaja K., Kim A., Lüscher B., Baron J.M. (2016). Control of the physical and antimicrobial skin barrier by an IL-31–IL-1 signaling network. J. Immunol..

[107] Simon M., Singh B., Jegga A.G., Shanmukhappa K.S., Edukulla R., Khurana G.H., Medvedovic M., Dillon S.R., Madala S.K. (2016). IL-31-driven skin remodeling involves epidermal cell proliferation and thickening that lead to impaired skin-barrier function. PLoS ONE.

[108] Cevikbas F., Wang X., Akiyama T., Kempkes C., Savinko T., Antal A., Kukova G., Buhl T., Ikoma A., Buddenkotte J. (2014). A sensory neuron-expressed IL-31 receptor mediates T helper cell-dependent itch: Involvement of TRPV1 and TRPA1. J. Allergy Clin. Immunol..

[109] Feld M., Garcia R., Buddenkotte J., Katayama S., Lewis K., Muirhead G., Hevezi P., Plesser K., Schrumpf H., Krjutskov K. (2016). The pruritus- and Th 2-associated cytokine IL-31 promotes growth of sensory nerves. J. Allergy Clin. Immunol..

[110] Hawro T., Saluja R., Weller K., Altrichter S., Metz M., Maurer M. (2014). Interleukin-31 does not induce immediate itch in atopic dermatitis patients and healthy controls after skin challenge. Allergy.

[111] Meng J., Moriyama M., Feld M., Buddenkotte J., Buhl T., Szöllösi A., Zhang J., Miller P., Ghetti A., Fischer M. (2018). New mechanism underlying IL-31–induced atopic dermatitis. J. Allergy Clin. Immunol..

[112] Richards C., Gandhi R., Botelho F., Ho L., Paolini J. (2020). Oncostatin M induction of monocyte chemoattractant protein 1 is inhibited by anti-oncostatin M receptor beta monoclonal antibody KPL-716. Acta Derm. Venereol..

[113] Rincón M.A.J., Nakamura T., Fikrig E., Flavell R.A. (1997). Interleukin (IL)-6 directs the differentiation of IL-4-producing CD4+ T cells. J. Exp. Med..

[114] Toshitani A., Ansel J.C., Chan S.C., Li S.H., Hanifin J.M. (1993). Increased interleukin 6 production by T cells derived from patients with atopic dermatitis. J. Investig. Derm..

[115] Conti P., Kempuraj D., Gioacchino M.D., Boucher W., Letourneau R., Kandere K., Barbacane R.C., Reale M., Felaco M., Frydas S. (2002). Interleukin-6 and mast cells. Allergy Asthma Proc..

[116] Fedenko E.S., Elisyutina O.G., Filimonova T.M., Boldyreva M.N., Burmenskaya O.V., Rebrova O.Y., Yarilin A.A., Khaitov R.M. (2011). Cytokine gene expression in the skin and peripheral blood of atopic dermatitis patients and healthy individuals. Self/Nonself.

[117] Navarini A.A., French L.E., Hofbauer G.F.L. (2011). Interrupting IL-6–receptor signaling improves atopic dermatitis but associates with bacterial superinfection. J. Allergy Clin. Immunol..

[118] Keshari S., Sipayung A.D., Hsieh C.C., Su L.J., Chiang Y.R., Chang H.C., Yang W.C., Chuang T.H., Chen C.L., Huang C.M. (2019). IL-6/p-BTK/P-ERK signaling mediates calcium phosphate-induced pruritus. FASEB J..

[119] Konda D., Chandrashekar L., Rajappa M., Kattimani S., Thappa D.M., Ananthanarayanan P.H. (2015). Serotonin and interleukin-6: Association with pruritus severity, sleep quality and depression severity in prurigo nodularis. Asian J. Psychiatr..

[120] Kido-Nakahara M., Buddenkotte J., Kempkes C., Ikoma A., Cevikbas F., Akiyama T., Nunes F., Seeliger S., Hasdemir B., Mess C. (2014). Neural peptidase endothelin-converting enzyme 1 regulates endothelin 1-induced pruritus. J. Clin. Investig..

[121] Aktar M.K., Kido-Nakahara M., Furue M., Nakahara T. (2015). Mutual upregulation of endothelin-1 and IL-25 in atopic dermatitis. Allergy.

[122] Tsybikov N.N., Petrisheva I.V., Kuznik B.I., Magen E. (2015). Plasma endothelin-1 levels during exacerbation of atopic dermatitis. Allergy Asthma Proc..

[123] Wong L.S., Yen Y.T., Lin S.H., Lee C.H. (2020). IL-17A induces endothelin-1 expression through p38 pathway in prurigo nodularis. J. Investig. Derm..

[124] Borowczyk J., Shutova M., Brembilla N.C., Boehncke W.-H. (2021). IL-25 (IL-17E) in epithelial immunology and pathophysiology. J. Allergy Clin. Immunol..

[125] Yamada Y., Matsumoto T. (2017). House dust mites induce production of endothelin-1 and matrix metalloproteinase-9 in keratinocytes via proteinase-activated receptor-2 activation. Int. Arch. Allergy Immunol..

[126] Kido-Nakahara M., Wang B., Ohno F., Tsuji G., Ulzii D., Takemura M., Furue M., Nakahara T. (2020). Inhibition of mite-induced dermatitis, pruritus, and nerve sprouting in mice by the endothelin receptor antagonist bosentan. Allergy.

[127] Nockher W.A., Renz H. (2006). Neurotrophins in allergic diseases: From neuronal growth factors to intercellular signaling molecules. J. Allergy Clin. Immunol..

[128] Dou Y.C., Hagströmer L., Emtestam L., Johansson O. (2006). Increased nerve growth factor and its receptors in atopic dermatitis: An immunohistochemical study. Arch. Derm..

[129] Toyoda M., Nakamura M., Makino T., Hino T., Kagoura M., Morohashi M. (2002). Nerve growth factor and substance P are useful plasma markers of disease activity in atopic dermatitis. Br. J. Derm..

[130] Papoiu A.D.P., Wang H., Nattkemper L., Tey H.L., Ishiuji Y., Chan Y.H., Schmelz M., Yosipovitch G. (2011). A study of serum concentrations and dermal levels of NGF in atopic dermatitis and healthy subjects. Neuropeptides.

[131] Schulte-Herbrüggen O., Fölster-Holst R., Von Elstermann M., Augustin M., Hellweg R. (2007). Clinical relevance of nerve growth factor serum levels in patients with atopic dermatitis and psoriasis. Int. Arch. Allergy Immunol..

[132] Roggenkamp D., Falkner S., Stäb F., Petersen M., Schmelz M., Neufang G. (2012). Atopic keratinocytes induce increased neurite outgrowth in a coculture model of porcine dorsal root ganglia neurons and human skin cells. J. Investig. Derm..

[133] Kritas S.K., Caraffa A., Antinolfi P., Saggini A.P.A., Rosati M., Tei M., Speziali A., Saggini R., Pandolfi F., Pantalone A. (2014). Nerve growth factor interactions with mast cells. Int. J. Immunopathol. Pharm..

[134] Kawakami T., Ando T., Kimura M., Wilson B.S., Kawakami Y. (2009). Mast cells in atopic dermatitis. Curr. Opin. Immunol..

[135] Fölster-Holst R., Papakonstantinou E., Rüdrich U., Buchner M., Pite H., Gehring M., Kapp A., Weidinger S., Raap U. (2016). Childhood atopic dermatitis-brain-derived neurotrophic factor correlates with serum eosinophil cationic protein and disease severity. Allergy.

[136] Raap U., Goltz C., Deneka N., Bruder M., Renz H., Kapp A., Wedi B. (2005). Brain-derived neurotrophic factor is increased in atopic dermatitis and modulates eosinophil functions compared with that seen in nonatopic subjects. J. Allergy Clin. Immunol..

[137] Guseva D., Rüdrich U., Kotnik N., Gehring M., Patsinakidis N., Agelopoulos K., Ständer S., Homey B., Kapp A., Gibbs B.F. (2020). Neuronal branching of sensory neurons is associated with BDNF-positive eosinophils in atopic dermatitis. Clin. Exp. Allergy.

[138] Roblin D., Yosipovitch G., Boyce B., Robinson J., Sandy J., Mainero V., Wickramasinghe R., Anand U., Anand P. (2015). Topical TrkA kinase inhibitor CT327 is an effective, novel therapy for the treatment of pruritus due to psoriasis: Results from experimental studies, and efficacy and safety of CT327 in a phase 2b clinical trial in patients with psoriasis. Acta Derm. Venereol..

[139] Mashaghi A., Marmalidou A., Tehrani M., Grace P.M., Pothoulakis C., Dana R. (2016). Neuropeptide substance P and the immune response. Cell. Mol. Life Sci..

[140] Meixiong J., Dong X.Z. (2017). Mas-related g protein-coupled receptors and the biology of itch sensation. Annu. Rev. Genet..

[141] Lönndahl L., Rasul A., Lonne-Rahm S.-B., Holst M., Johansson B., El-Nour H., Radu Djurfeldt D., Nordlind K. (2019). Tachykinin upregulation in atopic dermatitis. Immunopharmacol. Immunotoxicol..

[142] Paramita D.A., Nasution K., Lubis N.Z. (2021). Relationship of substance P with the degree of atopic dermatitis severity. Clin. Cosmet. Investig. Derm..

[143] Siiskonen H., Harvima I. (2019). Mast cells and sensory nerves contribute to neurogenic inflammation and pruritus in chronic skin inflammation. Front. Cell. Neurosci..

[144] Subramanian H., Gupta K., Ali H. (2016). Roles of Mas-related g protein–coupled receptor X2 on mast cell–mediated host defense, pseudoallergic drug reactions, and chronic inflammatory diseases. J. Allergy Clin. Immunol..

[145] Taracanova A., Tsilioni I., Conti P., Norwitz E.R., Leeman S.E., Theoharides T.C. (2018). Substance P and IL-33 administered together stimulate a marked secretion of IL-1β from human mast cells, inhibited by methoxyluteolin. Proc. Natl. Acad. Sci. USA.

[146] Taracanova A., Alevizos M., Karagkouni A., Weng Z., Norwitz E., Conti P., Leeman S.E., Theoharides T.C. (2017). SP and IL-33 together markedly enhance TNF synthesis and secretion from human mast cells mediated by the interaction of their receptors. Proc. Natl. Acad. Sci. USA.

[147] Shi X., Wang L., Clark J.D., Kingery W.S. (2013). Keratinocytes express cytokines and nerve growth factor in response to neuropeptide activation of the ERK1/2 and JNK MAPK transcription pathways. Regul. Pept..

[148] Andoh T., Nagasawa T., Satoh M., Kuraishi Y. (1998). Substance P induction of itch-associated response mediated by cutaneous NK1 tachykinin receptors in mice. J. Pharmacol. Exp. Ther..

[149] Azimi E., Reddy V.B., Shade K.-T.C., Anthony R.M., Talbot S., Pereira P.J.S., Lerner E.A. (2016). Dual action of neurokinin-1 antagonists on Mas-related GPCRS. JCI Insight.

[150] Azimi E., Reddy V.B., Pereira P.J.S., Talbot S., Woolf C.J., Lerner E.A. (2017). Substance P activates Mas-related G protein–coupled receptors to induce itch. J. Allergy Clin. Immunol..

[151] Akiyama T., Tominaga M., Davoodi A., Nagamine M., Blansit K., Horwitz A., Carstens M.I., Carstens E. (2013). Roles for substance P and gastrin-releasing peptide as neurotransmitters released by primary afferent pruriceptors. J. Neurophysiol..

[152] Sheahan T.D., Warwick C.A., Fanien L.G., Ross S.E. (2020). The neurokinin-1 receptor is expressed with gastrin-releasing peptide receptor in spinal interneurons and modulates itch. J. Neurosci..

[153] Lai J.-P., Cnaan A., Zhao H., Douglas S.D. (2008). Detection of full-length and truncated neurokinin-1 receptor mRNA expression in human brain regions. J. Neurosci. Methods.

[154] Ohmura T., Hayashi T., Satoh Y., Konomi A., Jung B., Satoh H. (2004). Involvement of substance P in scratching behaviour in an atopic dermatitis model. Eur. J. Pharm..

[155] Stander S., Siepmann D., Herrgott I., Sunderkotter C., Luger T.A. (2010). Targeting the neurokinin receptor 1 with aprepitant: A novel antipruritic strategy. PLoS ONE.

[156] Lönndahl L., Holst M., Bradley M., Killasli H., Heilborn J., Hall M., Theodorsson E., Holmberg J., Nordlind K. (2018). Substance P antagonist aprepitant shows no additive effect compared with standardized topical treatment alone in patients with atopic dermatitis. Acta. Derm. Venereol..

[157] Granstein R.D., Wagner J.A., Stohl L.L., Ding W. (2015). Calcitonin gene-related peptide: Key regulator of cutaneous immunity. Acta Physiol..

[158] Ding W.H., Stohl L.L., Wagner J.A., Granstein R.D. (2008). Calcitonin gene-related peptide biases Langerhans cells toward Th2-type immunity. J. Immunol..

[159] Järvikallio A., Harvima I.T., Naukkarinen A. (2003). Mast cells, nerves and neuropeptides in atopic dermatitis and nummular eczema. Arch. Derm. Res..

[160] Hodeib A., EI-Samad Z.A., Hanafy H., EI-Latief A.A., EI-Bendary A., Abu-Raya A. (2010). Nerve growth factor, neuropeptides and cutaneous nerves in atopic dermatitis. Indian J. Derm..

[161] McCoy E.S., Taylor-Blake B., Sarah S.E., Pribisko A.L., Zheng J., Zylka M.J. (2013). Peptidergic CGRPα primary sensory neurons encode heat and itch and tonically suppress sensitivity to cold. Neuron.

[162] Katsuno M., Aihara M., Kojima M., Osuna H., Hosoi J., Nakamura M., Toyoda M., Matsuda H., Ikezawa Z. (2003). Neuropeptides concentrations in the skin of a murine (NC/Nga mice) model of atopic dermatitis. J. Derm. Sci..

[163] Andoh T., Asakawa Y., Kuraishi Y. (2018). Non-myelinated C-fibers, but not myelinated α-fibers, elongate into the epidermis in dry skin with itch. Neurosci. Lett..

[164] Umemoto N., Kakurai M., Okazaki H., Kiyosawa T., Demitsu T., Nakagawa H. (2003). Serum levels of vasoactive intestinal peptide are elevated in patients with atopic dermatitis. J. Derm. Sci..

[165] Teresiak-Mikołajczak E., Czarnecka-Operacz M., Jenerowicz D., Silny W. (2013). Neurogenic markers of the inflammatory process in atopic dermatitis: Relation to the severity and pruritus. Pestepy Derm. Alergol..

[166] Ganea D., Hooper K.M., Kong W. (2015). The neuropeptide vasoactive intestinal peptide: Direct effects on immune cells and involvement in inflammatory and autoimmune diseases. Acta Physiol..

[167] Oda N., Miyahara N., Taniguchi A., Morichika D., Senoo S., Fujii U., Itano J., Gion Y., Kiura K., Kanehiro A. (2019). Requirement for neuropeptide Y in the development of type 2 responses and allergen-induced airway hyperresponsiveness and inflammation. Am. J. Physiol. Lung Cell Mol. Physiol..

[168] Lou H., Lu J., Choi E.B., Oh M.H., Jeong M., Barmettler S., Zhu Z., Zheng T. (2017). Expression of IL-22 in the skin causes Th2-biased immunity, epidermal barrier dysfunction, and pruritus via stimulating epithelial Th2 cytokines and the grp pathway. J. Immunol..

[169] Tirado-Sánchez A., Bonifaz A., Ponce-Olivera R.M. (2015). Serum gastrin-releasing peptide levels correlate with disease severity and pruritus in patients with atopic dermatitis. Br. J. Derm..

[170] Pagani M., Albisetti G.W., Sivakumar N., Wildner H., Santello M., Johannssen H.C., Zeilhofer H.U. (2019). How gastrin-releasing peptide opens the spinal gate for itch. Neuron.

[171] Vasiadi M., Mondolfi A.P., Alysandratos K.D., Therianou A., Katsarou-Katsari A., Petrakopoulou T., Theoharidis A., Miniati A., Theoharides T.C. (2013). Neurotensin serum levels and skin gene expression are increased in atopic dermatitis. Br. J. Derm..

[172] Hagströmer L., Emtestam L., Stridsberg M., Talme T. (2006). Expression pattern of somatostatin receptor subtypes 1-5 in human skin: An immunohistochemical study of healthy subjects and patients with psoriasis or atopic dermatitis. Exp. Derm..

[173] Sun L., Liu W., Zhang L. (2019). The role of Toll-like receptors in skin host defense, psoriasis, and atopic dermatitis. J. Immunol. Res..

[174] Zhang Y., Wang H.-C., Feng C., Yan M. (2018). Analysis of the association of polymorphisms rs5743708 in TLR2 and rs4986790 in TLR4 with atopic dermatitis risk. Immunol. Investig..

[175] Yu Y., Lin D., Cai X., Cui D., Fang R., Zhang W., Yu B., Wang X. (2020). Enhancement of chemokine mrna expression by toll-like receptor 2 stimulation in human peripheral blood mononuclear cells of patients with atopic dermatitis. Biomed. Res. Int..

[176] Yu Y., Zhang Y., Zhang J., Dou X., Yang H., Shao Y., Wang K., Yu B., Zhang W., Lau H.Y.A. (2015). Impaired Toll-like receptor 2-mediated Th1 and Th17/22 cytokines secretion in human peripheral blood mononuclear cells from patients with atopic dermatitis. J. Transl. Med..

[177] Nakamura N., Tamagawa-Mineoka R., Ueta M., Konishi E., Yasuike R., Masuda K., Matsunaka H., Murakami Y., Yokosawa E., Katoh N. (2019). Stratum corneum Toll-like receptor 3 expressions correlate with the severity of atopic dermatitis lesions. J. Derm. Sci..

[178] Szöllősi A.G., McDonald I., Szabó I.L., Meng J., van den Bogaard E., Steinhoff M. (2019). TLR3 in chronic human itch: A keratinocyte-associated mechanism of peripheral itch sensitization. J. Investig. Derm..

[179] Yasuike R., Tamagawa-Mineoka R., Ueta M., Nakamura N., Kinoshita S., Katoh N. (2017). The role of Toll-like receptor 3 in chronic contact hypersensitivity induced by repeated elicitation. J. Derm. Sci..

[180] Liu T., Berta T., Xu Z.-Z., Park C.-K., Zhang L., Lü N., Liu Q., Liu Y., Gao Y.-J., Liu Y.-C. (2012). TLR3 deficiency impairs spinal cord synaptic transmission, central sensitization, and pruritus in mice. J. Clin. Investig..

[181] Liu T., Xu Z.Z., Park C.K., Berta T., Ji R.R. (2010). Toll-like receptor 7 mediates pruritus. Nat. Neurosci..

[182] Liu T., Han Q., Chen G., Huang Y., Zhao L.-X., Berta T., Gao Y.-J., Ji R.-R. (2016). Toll-like receptor 4 contributes to chronic itch, alloknesis, and spinal astrocyte activation in male mice. Pain.

[183] Jia L., Lee S., Tierney J.A., Elmquist J.K., Burton M.D., Gautron L. (2021). TLR4 signaling selectively and directly promotes CGRP release from vagal afferents in the mouse. eNeuro.

[184] Kim S., Park G., Kim D., Lee J., Min H., Wall E., Lee C.J., Simon M.I., Lee S.J., Han S.K. (2011). Analysis of cellular and behavioral responses to imiquimod reveals a unique itch pathway in transient receptor potential vanilloid 1 (TRPV1)-expressing neurons. Proc. Natl. Acad. Sci. USA.

[185] Diaz A., Guttman-Yassky E. (2019). Topical agents for the treatment of atopic dermatitis. Expert Rev. Clin. Immunol..

[186] Lehto M., Savinko T., Wolff H., Kvist P.H., Kemp K., Lauerma A., Alenius H. (2010). A murine model of epicutaneous protein sensitization is useful to study efficacies of topical drugs in atopic dermatitis. Int. Immunopharmacol..

[187] Nakahara T., Morimoto H., Murakami N., Furue M. (2018). Mechanistic insights into topical tacrolimus for the treatment of atopic dermatitis. Pediatr. Allergy. Immunol..

[188] Luger T., Paller A.S., Irvine A.D., Sidbury R., Eichenfield L.F., Werfel T., Bieber T. (2021). Topical therapy of atopic dermatitis with a focus on pimecrolimus. J. Eur. Acad. Derm. Venereol..

[189] Reber L.L., Frossard N. (2014). Targeting mast cells in inflammatory diseases. Pharmacol. Ther..

[190] Paivandy A., Pejler G. (2021). Novel strategies to target mast cells in disease. J. Innate Immun..

[191] Siebenhaar F., Redegeld F.A., Bischoff S.C., Gibbs B.F., Maurer M. (2018). Mast cells as drivers of disease and therapeutic targets. Trends Immunol..

[192] Jeong H.-J., Ryu K.-J., Kim H.-M. (2018). Anticancer agent ABT-737 possesses anti-atopic dermatitis activity via blockade of caspase-1 in atopic dermatitis in vitro and in vivo models. Immunopharmacol. Immunotoxicol..

[193] Alvarez-Twose I., Matito A., Morgado J.M., Sánchez-Muñoz L., Jara-Acevedo M., García-Montero A., Mayado A., Caldas C.T.C., Muñoz-González J.I., Mollejo M. (2017). Imatinib in systemic mastocytosis: A phase IV clinical trial in patients lacking exon 17 kit mutations and review of the literature. Oncotarget.

[194] Hu X., Sang Y., Yang M., Chen X., Tang W. (2018). Prevalence of chronic kidney disease-associated pruritus among adult dialysis patients. Medicine.

[195] Martin C.E., Clotet-Freixas S., Farragher J.F., Hundemer G.L. (2020). Have we just scratched the surface? A narrative review of uremic pruritus in 2020. Can. J. Kidney Health Dis..

[196] Simonsen E., Komenda P., Lerner B., Askin N., Bohm C., Shaw J., Tangri N., Rigatto C. (2017). Treatment of uremic pruritus: A systematic review. Am. J. Kidney. Dis..

[197] Silverberg J.I., Brieva J. (2019). A successful case of dupilumab treatment for severe uremic pruritus. JAAD Case Rep..

[198] Oweis A.O., Al-Qarqaz F., Bodoor K., Heis L., Alfaqih M.A., Almomani R., Obeidat M.A., Alshelleh S.A. (2021). Elevated interleukin 31 serum levels in hemodialysis patients are associated with uremic pruritus. Cytokine.

[199] Patel S.P., Vasavda C., Ho B., Meixiong J., Dong X., Kwatra S.G. (2019). Cholestatic pruritus: Emerging mechanisms and therapeutics. J. Am. Acad. Derm..

